# Nomogram for predicting the likelihood of liver metastases at initial diagnosis in patients with Siewert type II gastroesophageal junction adenocarcinoma

**DOI:** 10.1038/s41598-023-37318-3

**Published:** 2023-07-07

**Authors:** Min Zhang, Wenwen Yang, Yanjiang Yang, Chengfeng Cai, Dan Zhao, Biao Han

**Affiliations:** 1 The First Clinical Medical College, Lanzhou University, Lanzhou, 730000 Gansu Province China; 2 Department of Thoracic Surgery, The First Hospital of Lanzhou University, Lanzhou, 730000 Gansu Province China; 3 Gansu Province International Cooperation Base for Research and Application of Key Technology of Thoracic Surgery, The First Hospital of Lanzhou University, Lanzhou, 730000 Gansu Province China; 4 Qilu Hospital of Shandong University, Shandong University, Jinan, 250355 Shandong Province China; 5 Department of Urology, Shaoxing People’s Hospital (Shaoxing Hospital, Zhejiang University School of Medicine), No. 568, Zhongxing North Road, Shaoxing, 312000 Zhejiang China; 6 Gansu University of Chinese Medicine, Lanzhou, 730000 China

**Keywords:** Cancer, Cancer screening

## Abstract

The liver is one of the most ordinary metastatic sites of gastroesophageal junction adenocarcinoma and significantly affects its prognosis. Therefore, this study tried to construct a nomogram that can be applied to predict the likelihood of liver metastases from gastroesophageal junction adenocarcinoma. 3001 eligible patients diagnosed with gastroesophageal junction adenocarcinoma between 2010 and 2015 in the Surveillance, Epidemiology, and End Results (SEER) database were involved in the analysis. Patients were randomly divided into a training cohort and an internal validation cohort using R software, with an allocation ratio of 7:3. According to the consequences of univariate and multivariate logistic regression, we constructed a nomogram for predicting the risk of liver metastases. The discrimination and calibration ability of the nomogram was appraised by the C-index, ROC curve, calibration plots, and decision curve analysis (DCA). We also used Kaplan–Meier survival curves to compare differences in overall survival in patients with gastroesophageal junction adenocarcinoma with and without liver metastases. Liver metastases developed in 281 of 3001 eligible patients. The overall survival of patients with gastroesophageal junction adenocarcinoma with liver metastases before and after propensity score matching (PSM) was obviously lower than that of patients without liver metastases. Six risk factors were finally recognized by multivariate logistic regression, and a nomogram was constructed. The C-index was 0.816 in the training cohort and 0.771 in the validation cohort, demonstrating the good predictive capacity of the nomogram. The ROC curve, calibration curve, and decision curve analysis further demonstrated the good performance of the predictive model. The nomogram can accurately predict the likelihood of liver metastases in gastroesophageal junction adenocarcinoma patients.

## Introduction

The incidence of gastroesophageal junction adenocarcinoma (GEJA) has increased markedly in Western countries over the past few decades^1,2^. Gastroesophageal junction cancer is a cancer in which the center of the tumor is situated in the gastroesophageal junction region. The widely used categorization of gastroesophageal junction cancer in Western countries is the Siewert classification^3^. The Siewert classification applies only to adenocarcinomas situated within 5 cm above or below the gastroesophageal junction. The Siewert classification divided GEJA into three types: type I is located about 1–5 cm above the esophagogastric junction; type II is located between 1 cm above and 2 cm below the esophagogastric junction; type III is located about 2–5 cm below the esophagogastric junction^4,5^. Cells at the gastroesophageal junction have histological features of both esophageal and gastric cells^6^. Therefore, its histological origin and appropriate treatment remain controversial^7,8^. In clinical practice, type I and type III GEJA are often treated and staged with reference to esophageal and gastric cancers^9^. Siewert type II GEJA is located along the borderline between the mediastinum and abdomen, they can metastasize to both thoracic and abdominal cavities. Therefore, the prognosis or metastasis of Siewert type II GEJA could be significantly different from other types of GEJA^10–12^. A previous study found that the poor prognosis of GEJA was largely attributable to early and frequent metastases^13^. Gastroesophageal junction carcinoma patients have a poor prognosis after metastases, with a 5-year survival rate of about 11%^14^. A population-based study showed that the liver is the most common site of metastasis for Siewert type II GEJA^15^. Consequently, it has important clinical value to construct a predictive model that can be applied to predict the risk of liver metastases from GEJA. This study tried to construct and validate a nomogram based on the SEER database for predicting the likelihood of liver metastases from Siewert type II GEJA.

## Methods

### Patients

**Figure 1:**
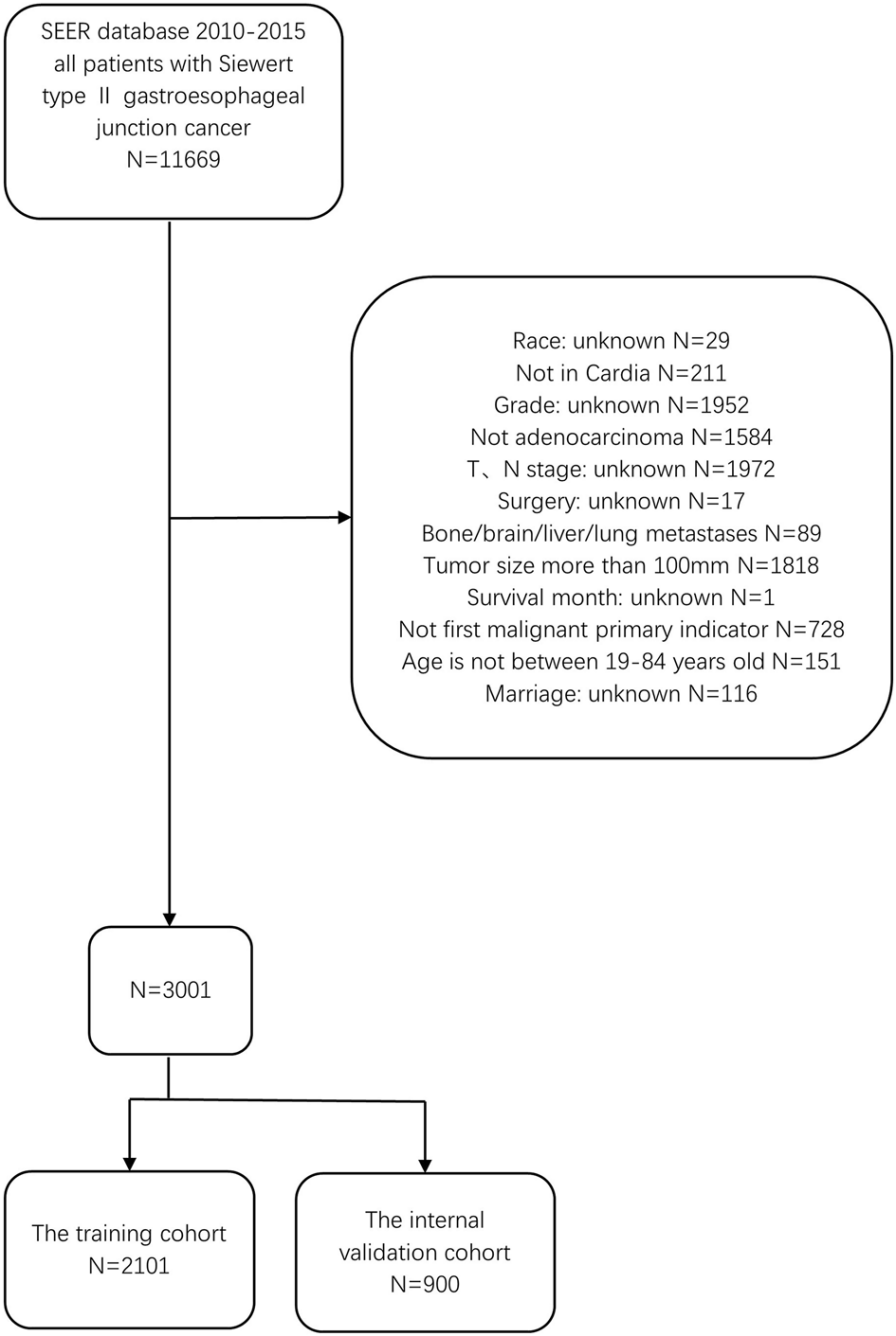
Patient screening flowchart. This figure contains how we screened 3001 Siewert type II gastroesophageal junction adenocarcinoma patients from the SEER database.

We screened 3001 GEJA patients newly diagnosed between 2010 and 2015 from the SEER database who met our inclusion criteria, of which 281 developed liver metastases. The exclusion process is shown in Fig. 1. Patients included in this study must meet the following criteria: (1) Tumor size does not exceed 100 mm (2) First malignant primary indicator (3) Age between 19 and 84 (4) The pathological type is adenocarcinoma. The exclusion criteria for GEJA patients were as follows: (1) Incomplete clinical and pathological features. (2) Patients who were identified by autopsy. We extracted race, gender, year of diagnosis, T stage, N stage, tumor size, age, and bone/brain/liver/lung metastases, as well as other follow-up data, from the SEER database. This study adopted the 7th edition of the American Joint Committee on Cancer (AJCC) TNM staging. All data used in this study were anonymized and de-identified from the SEER database. Therefore, approval by an institutional review board is not required, nor is informed consent of all subjects and/or their legal guardian(s). All methods of this study were performed in accordance with the relevant regulations and guidelines.

### Statistical analysis and optimal cutoffs

We used x-tile v3.6.1 (Yale University) software to determine optimal cutoff values for tumor size and age^16^. By using X-tile, we transform all continuous variables into categorical variables. We used Fisher's exact test or chi-square to compare differences in categorical variables. To balance differences in other factors between GEJA patients with liver metastases and those without liver metastases, we performed a 1:3 PSM in R software v4.3.0. The Kaplan–Meier survival curves were applied to evaluate the difference in survival time between patients with liver metastases and those without liver metastases before and after PSM. We randomly divided the 3001 GEJA patients into a training group (n = 2101) and an internal validation group (n = 900) in a 7:3 ratio by R software. We performed univariate and multivariate logistic regression, from which we created a nomogram for predicting the possibility of liver metastases. We use ROC curve, C-index, calibration curve, and decision curve analysis to verify its validity. All statistical analyses were carried out using GraphPad Prism v8.0.2 (GraphPad Software, Inc.), SPSS v26.0 (SPSS Inc.), and R software v4.1.3 (https://www.r-project.org/). The difference is statistically significant at *P* < 0.05.

## Results

### Characteristics of GEJA patients

We included 3001 patients with GEJA diagnosed between 2010 and 2015 in this retrospective study, with 9.3% (n = 281) had liver metastases, 3.5% (n = 106) had lung metastases, 2.5% (n = 77) had bone metastases, 0.4% (n = 14) had brain metastases. Table 1 summarizes patient characteristics before and after PSM. As shown in Table 1, most of the variables have been balanced after PSM.

**Table 1:** Characteristics of all 3001 patients before and after PSM.

	the pre-PSM cohort					the post-PSM cohort				
	liver metastasis		Non-liver mestasis		*P*	liver metastasis		Non-liver mestasis		*P*
	N	%	N	%		N	%	N	%	
	n = 281		n = 2720			n = 221		n = 471		
Age										
20–66	183	65.12	1477	54.30	< 0.001	140	63.35	273	57.96	0.371
67–77	85	30.25	903	33.20		68	30.77	162	34.30	
78–84	13	4.63	340	12.50		13	5.88	36	7.64	
Race										
White	251	89.32	2403	88.35	0.103	197	89.14	422	89.60	0.903
Black	18	6.41	126	4.63		12	5.43	27	5.73	
Other	12	4.27	191	7.02		12	5.43	22	4.67	
Gender										
Female	44	15.66	511	18.79	0.226	35	15.84	67	14.23	0.658
Male	237	84.34	2209	81.21		186	84.16	404	85.77	
Grade										
Grade I	13	4.63	218	8.01	0.037	11	4.98	15	3.18	0.711
Grade II	106	37.72	1127	41.43		82	37.10	176	37.37	
Grade III	159	56.58	1327	48.79		126	57.01	275	58.39	
Grade IV	3	1.07	48	1.76		2	0.90	5	1.06	
T stage										
T1	123	43.77	702	25.81	< 0.001	89	40.27	148	31.42	0.087
T2	11	3.91	385	14.15		9	4.07	21	4.46	
T3	98	34.88	1438	52.87		90	40.72	237	50.32	
T4	49	17.44	195	7.17		33	14.93	65	13.80	
N stage										
N0	83	29.54	1116	41.03	< 0.001	64	28.96	125	26.54	0.693
N1	140	49.82	1002	36.84		111	50.23	233	49.97	
N2	38	13.52	390	14.34		30	13.57	80	16.99	
N3	20	7.12	212	7.79		16	6.88	33	7.01	
Surgery										
No	256	91.10	798	29.34	< 0.001	196	88.69	400	84.93	0.224
Yes	25	8.90	1922	70.66		25	11.31	71	15.07	
Radiation										
No	181	64.41	1152	42.35	< 0.001	129	58.37	224	47.56	1.010
Yes	100	35.59	1568	57.65		92	41.63	247	52.44	
Chemotherap										
No	64	22.78	781	28.71	0.037	53	23.98	91	19.32	0.191
Yes	217	77.22	1939	71.29		168	76.02	380	80.68	
Bone metastasis										
No	245	87.19	2679	98.49	< 0.001	198	89.59	442	93.84	0.068
Yes	36	12.81	41	1.51		23	10.41	29	6.17	
Brain metastasis										
No	275	97.86	2712	99.71	0.001	218	98.64	466	98.94	1.000
Yes	6	2.14	8	0.29		3	1.36	5	1.06	
Lung metastasis										
No	229	81.49	2666	98.01	< 0.001	194	87.78	433	91.93	0.109
Yes	52	18.51	54	1.99		27	12.22	38	8.07	
Tumor size										
1–19	13	4.63	526	19.34	< 0.001	13	5.88	18	3.82	0.421
20–42	105	37.37	1202	44.19		91	41.18	189	40.13	
43–100	163	58.01	992	36.47		117	52.94	264	56.05	
Marital status										
Unmarried	104	37.0107	849	31.21	0.051	78	35.29	161	34.18	0.841
Married	177	62.9893	1871	68.79		143	64.71	310	65.82	

We used the x-tile v3.6.1 (Yale University) to determine the optimal cutoffs for tumor size and age. PSM:propensity score matching.

### Survival analysis of liver metastases from gastroesophageal junction adenocarcinoma

**Figure 2:**
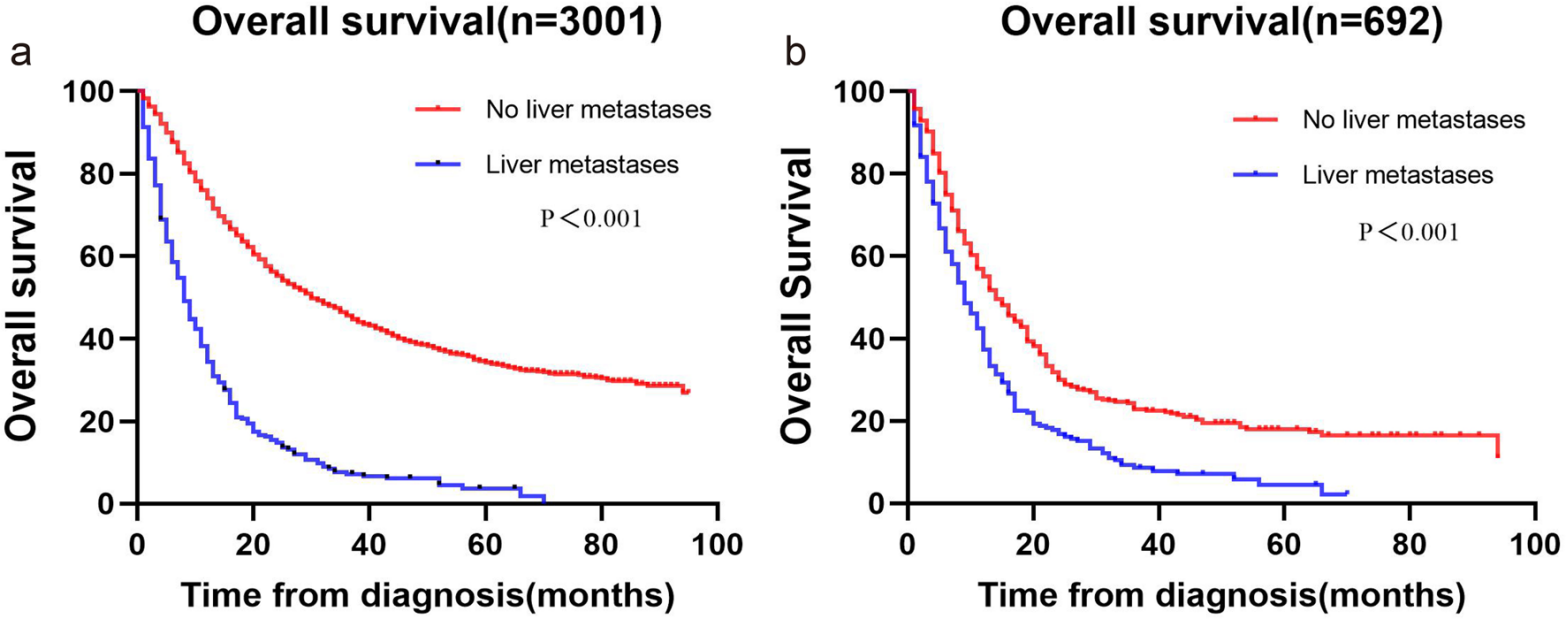
Kaplan–Meier curves of the pre- and post-PSM cohort. Kalpan–Meier curves of (**a**) the pre-PSM cohort (**b**) the post-PSM cohort.

Using R software v4.3.0, we performed a 1:3 propensity score matching of patients with gastroesophageal junction adenocarcinoma based on the presence or absence of liver metastases, and finally, 221 patients who had liver metastases were matched with 471 patients without liver metastases. The median follow-up for the pre-and post-PSM cohorts was 22 months (interquartile range: 10–44 months) and 11 months (interquartile range: 5–22 months), respectively. As shown in Table 1, other variables had been largely balanced. 1952 (65.0%) and 603 (87.1%) patients died during follow-up in the pre-and post-PSM cohorts, respectively. GEJA patients with liver metastases and those without liver metastases had median OS of 8.0 (95% CI 6.7–9.3) months and 30.0 (95% CI 27.7–32.3) months, respectively, in the pre-PSM cohort. (Fig. 2a) In the post-PSM cohort, they were 9.0 months (95% CI 6.5–9.5) and 14.0 months (95% CI 10.3–15.1), respectively. (Fig. 2b).

### The diagnostic likelihood of liver metastases in GEJA patients

We randomly divided the patients into a training cohort and an internal validation cohort with an allocation of 7:3 ratio by R software v4.3.0. More information about the training and validation cohorts is shown in Table 2. As shown in Table 3, we performed univariate and multivariate logistic regression in the training cohort using SPSS v26.0 and finally identified age, T stage, bone metastasis, lung metastasis, and tumor size as risk factors for liver metastasis in GEJA patients.

**Table 2:** Patient characteristics of the training and validation cohorts.

	The training cohort		The validation cohort		*P*
	N	%	N	%	
	n = 2101		n = 900		
Age					
20–66	1159	55.3	501	+	0.959
67–77	693	33.0	295	32.8	
78–84	249	11.9	104	11.68	
Race					
White	1862	88.6	792	88.0	0.206
Black	92	4.4	52	5.8	
Other	147	7	56	6.2	
Gender					
Female	372	17.7	183	20.3	0.099
Male	1729	82.3	717	79.7	
Grade					
Grade I	155	7.4	76	8.4	0.659
Grade II	857	40.8	376	41.8	
Grade III	1053	50.1	433	48.1	
Grade IV	36	1.7	15	1.7	
T stage					
T1	581	27.7	244	27.1	0.950
T2	280	13.3	116	12.9	
T3	1071	51.0	464	51.6	
T4	168	8.0	76	8.66	
N stage					
N0	841	40.0	358	39.8	0.728
N1	805	38.3	337	37.4	
N2	290	13.8	138	15.3	
N3	165	7.9	67	7.4	
Surgery					
No	744	35.4	311	33.6	0.284
Yes	1357	64.6	598	66.4	
Radiation					
No	872	41.5	438	48.7	0.008
Yes	1229	58.5	462	51.3	
Chemotherapy					
No	601	28.6	150	32.1	0.215
Yes	1500	71.4	289	67.9	
Bone metastasis					
No	2049	97.5	875	97.2	0.347
Yes	52	2.5	25	2.8	
Brain metastasis					
No	2094	99.7	893	99.2	0.145
Yes	7	0.3	7	0.8	
Lung metastasis					
No	2030	96.6	865	96.1	0.559
Yes	71	3.4	35	3.9	
Tumor size					
1–19	373	17.8	166	18.4	0.269
20–42	935	44.5	372	41.3	
43–100	793	37.7	362	40.2	
Marital status					
Unmarried	669	31.8	284	31.6	0.911
Married	1432	68.2	616	68.4	

We used the x-tile v3.6.1 (Yale University) to determine the optimal cutoffs for tumor size and age.

**Table 3:** Univariate and multivariate logistic regression for analyzing associated factors for developing liver metastases.

	Univariate		Multivariate	
	HR(95%CI)	*P*	HR(95%CI)	*P*
Age				
20–66	1	0.001	1	0.001
67–77	3.489(1.751–6.952)	0.001	0.787(0.555–1.117)	0.180
78–84	2.646(1.295–5.408)	0.008	0.283(0.139–0.578)	< 0.001
Race				
White	1	0.449	1	0.335
Black	1.112(0.567–2.182)	0.757	0.949(0.452–1.994)	0.891
Other	0.666(0.344–1.288)	0.227	0.582(0.284–1.193)	0.139
Sex				
Female	1			
Male	1.276(0.851–1.912)	0.238		
Grade				
Well differentiated; Grade I	1	0.035	1	0.116
Moderately differentiated; Grade II	1.183(0.612–2.287)	0.618	1.101(0.540–2.241)	0.792
Poorly differentiated; Grade III	1.700(0.895–3.2292)	0.105	1.391(0.690–2.802)	0.357
Undifferentiated; anaplastic; Grade IV	0.374(0.047–2.994)	0.354	0.156(0.017–1.454)	0.103
T stage				
T1	1	< 0.001	1	< 0.001
T2	0.098(0.039–0.244)	< 0.001	0.071(0.028–0.182)	< 0.001
T3	0.393(0.284–0.545)	< 0.001	0.206(0.138–0.308)	< 0.001
T4	1.417(0.918–2.188)	0.116	0.581(0.346–0.975)	0.040
N stage				
N0	1	0.007	1	0.084
N1	1.890(1.375–2.598)	0.001	1.532(1.031–2.277)	0.0039
N2	1.442(0.932–2.21)	0.505	1.333(0.758–2.342)	0.318
N3	1.273 (0.721–2.249)	0.840	0.815(0.395–1.683)	0.581
Bone metastasis				
No	1		1	
Yes	8.900(5.052–15.679)	< 0.001	4.219(2.216–8.032)	< 0.001
Lung metastasis				
No	1		1	
Yes	10.054(6.151–16.434)	< 0.001	6.401(3.649–11.226)	< 0.001
Brain metastasis				
No	1		1	
Yes	7.073(1.570–31.783)	0.011	2.189(0.268–17.856)	0.465
Marital status				
Unmarried	1			
Married	0.816(0.614–1.085)	0.163		
Tumor size				
1–19	1	< 0.001	1	< 0.001
20–42	3.940(1.959–7.924)	< 0.001	5.334(2.557–11.124)	< 0.001
43–100`	3.940(1.959–7.924)	< 0.001	8.576(4.049–18.165)	< 0.001

HR: hazard ratio; CI: confidence interval.

### Construction and validation of a predicted nomogram

**Figure 3:**
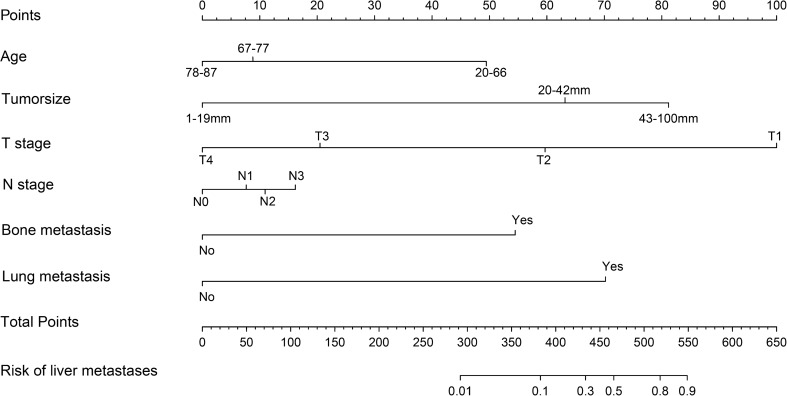
Nomogram to predict the risk of liver metastases in gastroesophageal junction adenocarcinoma patients. From this nomogram, we can determine the risk of liver metastasis of gastroesophageal junction adenocarcinoma.

**Figure 4:**
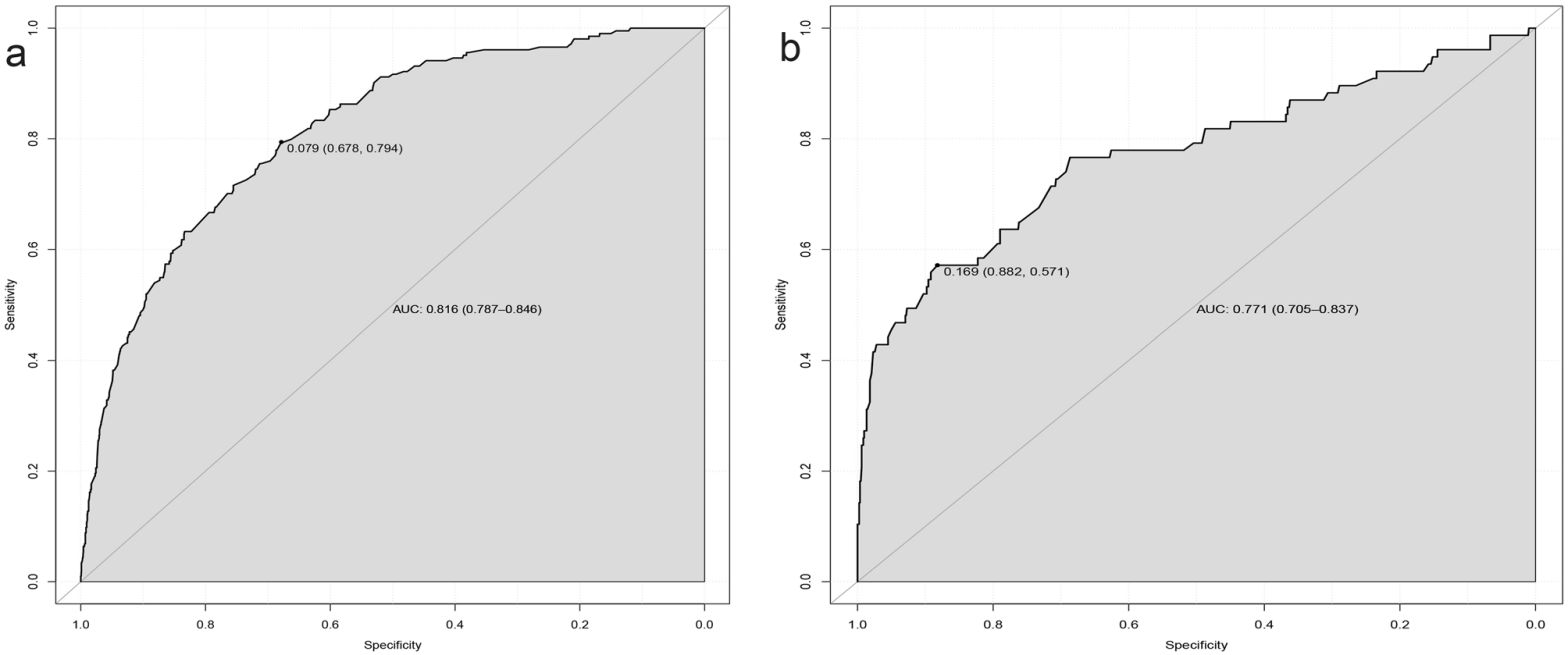
ROC curves of the nomogram. ROC curves of the nomogram in the training cohort (**a**) and the internal validation cohort (**b**).

**Figure 5:**
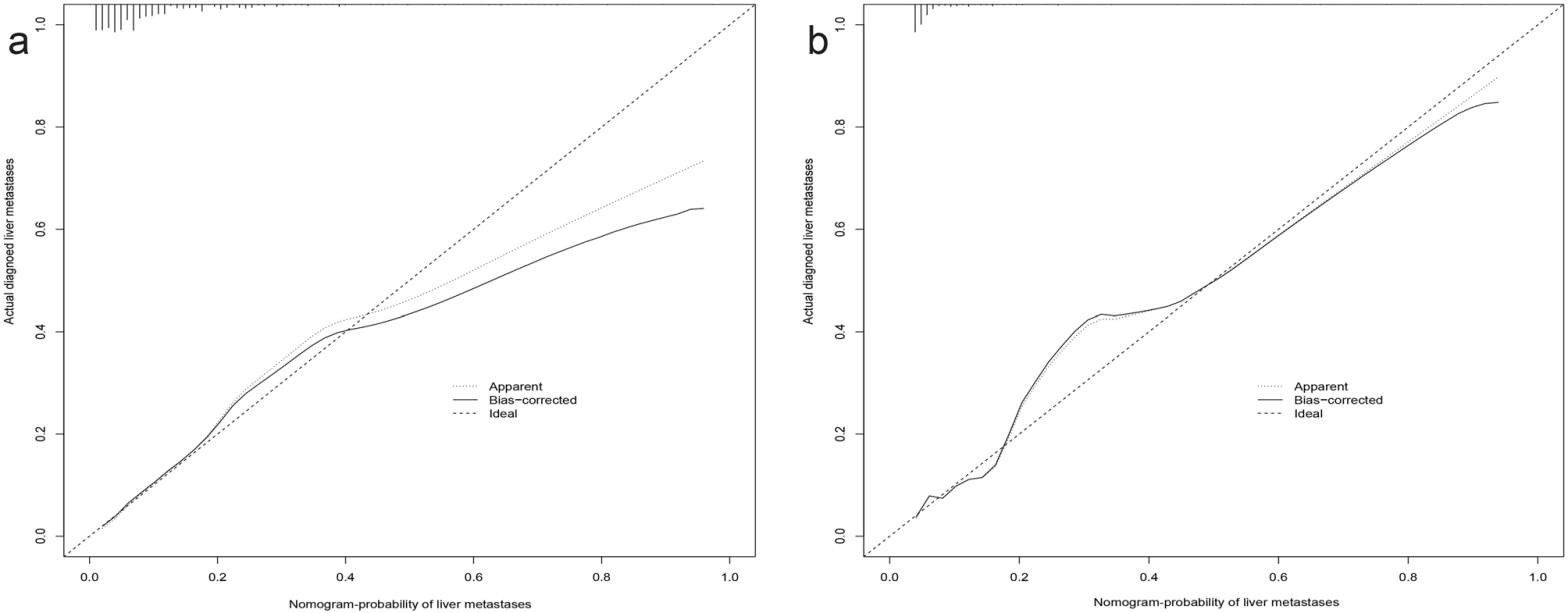
calibration curves of the nomogram. The calibration curves of the nomogram for gastroesophageal junction adenocarcinoma patients in the training cohort (**a**), and the internal validation cohort (**b**).

**Figure 6:**
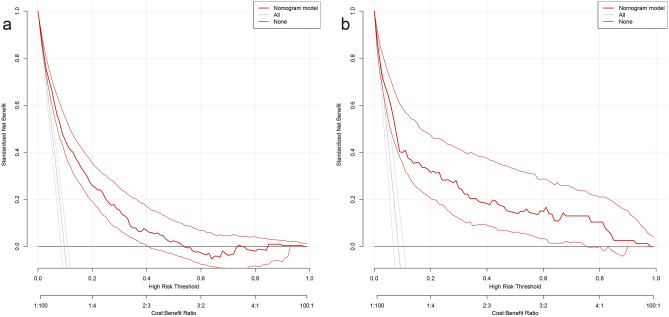
Evaluation of decision analysis (DCA) curves for nomination charts decision analysis curves (DCA) for patients with adenocarcinoma of the gastroesophageal junction in the training cohort (**a**) and the internal validation cohort (**b**).

Based on the risk factors for liver metastases identified by multivariate logistic regression, we created a nomogram to predict the risk of liver metastases in GEJA patients. (Fig. 3). The C-index in the training cohort is 0.816 and the C-index in the validation cohort is 0.771, which indicates that the prediction model has good discriminative ability as well as accuracy. As shown in Fig. 4, the AUC values of the nomogram in the training and validation cohorts are 0.816 and 0.771, respectively, which reflect the good predictive ability of our constructed prediction model. The calibration curves for both the training cohort (Fig. 5a) and the validation cohort (Fig. 5b) showed a good correlation between the predicted possibility of liver metastases and actually diagnosed liver metastases. A DCA was performed to determine the clinical utility of the nomogram. Figure 6 showed good positive net benefit in both the training cohort (Fig. 6a) and the internal validation cohort (Fig. 6b), indicating the good clinical applicability of the nomogram in predicting the presence of liver metastases in patients with adenocarcinoma of the gastroesophageal junction.

## Discussion

The survival of patients with metastatic Siewert type II GEJA is influenced by multiple factors, such as pathological type, age, metastatic pattern, degree of differentiation, and treatment^17,18^. The liver is the most ordinary site of distant metastases in patients with Siewert type II GEJA, and it is also the most prognostic factor in all distant metastases^19^. The liver is also the most ordinary metastatic site of esophageal and gastric cancers^20,21^. Once metastasis occurs, the prognosis of GEJA patients will be poor. Surgery is the most common method of treatment for GEJA. However, once liver metastasis occurs, the comprehensive benefit of surgery for patients will be very low, and radiotherapy and chemotherapy will become the first treatment for patients. We found that 9.3% (n = 281) of GEJA patients included in this study developed liver metastases, and 18.3% (n = 551) of GEJA patients developed distant metastases. The survival rate of GEJA patients with liver metastases was obviously lower than those without liver metastases. For patients with liver metastases, early detection and early treatment can greatly improve their survival time and quality of life. Ultrasound, CT, and PET-CT are commonly used methods to detect distant metastases in GEJA, and PET-CT can accurately exclude distant metastases^22,23^. However, it has been shown that 15–20% of new esophageal cancers have distant metastases that are not identified by CT, the sensitivity and specificity of detection of distant metastasis by CT are 52% and 91%, respectively^24,25^. In addition, these imaging tests have radiation hazards and high prices, and not every patient can afford to undergo long-term or frequent imaging tests. Therefore, constructing a nomogram to predict the risk of liver metastases in GEJA patients can better guide clinical practice. Nomograms have long been widely used in oncology because they can provide visual predictions of patient outcomes based on relevant clinical variables^26^. The results obtained through data analysis can demonstrate the high predictive performance as well as clinical utility of nomograms, which can reduce examination costs on the one hand and avoid radiation hazards on the other. Besides, nomograms can be used as an early and low-cost screening tool for tumor metastasis, which can be a very meaningful guide for tumor diagnosis, treatment, and prognosis. Several nomograms have previously been used to predict prognosis in patients with gastroesophageal junction cancer. However, a nomogram to predict the likelihood of liver metastases from gastroesophageal junction adenocarcinoma has not been constructed^6,27,28^. Therefore, based on the SEER database, we constructed a nomogram that can be used to predict the likelihood of liver metastases in GEJA patients.

In this retrospective study, a nomogram that can predict the risk of liver metastases resulting from GEJA was constructed. And its accuracy was verified by the ROC curve, C index, and calibration curve. Through univariate and multivariate logistic regression, we finally identified age, tumor size, N stage, T stage, bone metastases, and lung metastases as factors affecting liver metastases in gastroesophageal junction adenocarcinoma.

We found that the likelihood of liver metastases in GEJA patients decreased with increasing age. A previous study found that colorectal cancer incidence increases with age, but metastatic spread decreases with age^29,30^. And they further analyzed the possible mechanism and believed that this conclusion is the result of the interaction between the tumor microenvironment, tumor biology, the immune system, and the genome^30^. This is consistent with our conclusion that age is an important factor affecting cancer spread, and the risk of cancer metastasis decreases with age.

We found that tumor size was also an important factor affecting the happening of liver metastases in GEJA patients, and the larger the tumor size, the higher the risk of liver metastases. It has been previously reported that the risk of lymph node metastasis increases with increasing tumor size in patients with Siewert type II T1-T3 GEJA^31^. It can be seen that tumor size is related to tumor invasion, which is further demonstrated by our findings.

As shown in Fig. 3, N staging is the least influential among the many factors that affect the occurrence of liver metastases in GEJA. Previous studies have found that in non-small cell lung cancer, the rate of multiorgan metastases increases with increasing N stage^32^. Therefore, for GEJA patients, a higher N stage is linked with a higher risk of liver metastases. The mechanism behind it remains to be revealed in future studies.

T staging of gastroesophageal junction malignancies is based on the degree of invasion. However, to our surprise, we found that the higher the T stage, the lower the risk of liver metastases in GEJA patients. We also found that the risk of liver metastases in gastric cancer decreased with increasing T stage. Nevertheless, more evidence is needed to confirm the relationship between the T stage and liver metastases in GEJA patients.

Malignant tumors can metastasize to other organs in the body through blood spread. Therefore, it is not surprising that there is a correlation between bone metastases and lung metastases, and liver metastases. However, we found no correlation between brain and liver metastases in our study (*P* = 0.108). We believe this is due to the low number of patients with brain metastases in the patients included in this study (n = 14, 0.4%).

Therefore, age, tumor size, T stage, N stage, lung metastasis, and brain metastasis all affect the occurrence of liver metastases in GEJA patients. The nomogram we constructed can accurately predict the likelihood of liver metastases in GEJA patients and better guide clinical practice.

## Conclusion

The survival time of GEJA patients with liver metastases was obviously lower than that of GEJA patients without liver metastases. The nomogram model developed in our study can precisely predict the possibility of GEJA patients with liver metastases.

## Data Availability

The datasets used and/or analyzed during the current study available from the corresponding author on reasonable request. It can also be downloaded directly from the SEER database. (https://seer.cancer.gov/).
